# Advances in alginate biosynthesis: regulation and production in *Azotobacter vinelandii*

**DOI:** 10.3389/fbioe.2025.1593893

**Published:** 2025-07-31

**Authors:** Belén Ponce, Agustín Zamora-Quiroz, Ernesto González, Rodrigo Andler, Alvaro Díaz-Barrera

**Affiliations:** ^1^ Escuela de Ingeniería Bioquímica, Pontificia Universidad Católica de Valparaíso, Valparaíso, Chile; ^2^ Universidad Técnica Federico Santa María, Valparaíso, Chile; ^3^ Departamento de Ingeniería Química y de Materiales, Facultad de Ciencias Químicas, Universidad Complutense de Madrid, Madrid, Spain; ^4^ Escuela de Ingeniería en Biotecnología, Universidad Católica del Maule, Talca, Chile

**Keywords:** alginate, *Azotobacter vinelandii*, biosynthesis, regulation, oxygen transfer rate

## Abstract

Alginates are polysaccharides composed of (1–4)-β-D-mannuronic acid (M) and α-L-guluronic acid (G), whose proportions influence their rheological properties and a wide range of applications in the food, pharmaceutical, and biomedical industries. *Azotobacter vinelandii*, a Gram-negative bacterium, has been studied for its ability to produce alginate due to its capacity to fix atmospheric nitrogen and its high respiratory activity. The biosynthesis of alginate in *Azotobacter vinelandii* involves precursor synthesis, polymerization, modification, and secretion, which are regulated by complex mechanisms, including the secondary messenger c-di-GMP. This regulatory network links cellular respiration with alginate yield and molecular characteristics. Fermentation strategies show that high oxygen transfer rates (OTRs) enhance alginate production, whereas low OTRs favor the synthesis of alginate with higher molecular weights and higher G/M ratios, which are crucial for advanced applications such as hydrogels and drug delivery systems. Insights into these biosynthetic and regulatory processes enable scalable production of high-quality alginate, bridging laboratory research with industrial applications and expanding its potential in the biotechnological and medical fields.

## Introduction

Alginates are linear biopolymers present in the cell walls of brown algae and are produced by different bacteria, such as *Pseudomonas* spp. and *Azotobacter* sp. (Zhang et al., 2022; Hendawy et al., 2019; Bonartseva et al., 2017). These bacteria produce alginate as an exopolysaccharide in response to certain physiological processes, such as surface/cell adhesion, resistance to cytotoxic compounds, and dormant cell differentiation. *Azotobacter vinelandii* can also produce this biopolymer under vegetative growth conditions (Núñez et al., 2022; Ma et al., 2022; Castillo et al., 2013). The U.S. Food and Drug Administration (FDA) has cataloged the safe use of sodium alginate obtained from brown algae for diverse applications. Alginate can be used in the food, pharmaceutical, and cosmetic industries because of its high biocompatibility and advantageous physical–chemical properties, which encourage its use as a stabilizer and viscosifier agent, in addition to its ability to form a strong and stable hydrogel when interacting with divalent ions such as calcium (Hoefer et al., 2015).

**FIGURE 1:**
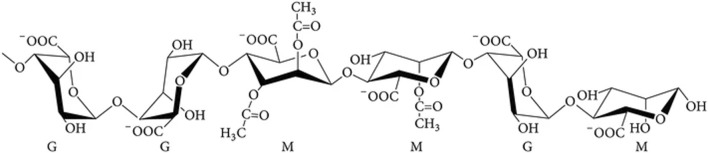
Chemical composition of bacterial alginate. Mannuronic acid (M) and guluronic acid (G) residues in the polymer.

Alginate is chemically composed of two monomers, β-D mannuronic acid (M) and its C-5 epimer α-L guluronic acid (G) (Figure 1). Its structure and conformation are influenced by the linking of these monomers in different sequences, forming patterns called blocks (Bojorges et al., 2023; Sanchez-Ballester et al., 2021; Hecht and Srebnik, 2016). M-residues can be acetylated on C-2 and/or C-3 during alginate synthesis in bacteria, which prevents the action of alginate-modifying enzymes (Cheng et al., 2020; Hecht and Srebnik, 2016). The chemical composition of alginate determines its properties and, therefore, its possible applications; for example, alginate with a relatively high molecular weight (between 400 and 3,420 kDa) has a higher viscosifying capacity, which is favorable for its use as a food additive (Hoefer et al., 2015; Schmid and Picker-Freyer, 2009). In the case of biomedical applications, a higher G/M ratio (with a ratio from 2.3 to 9) is associated with the presence of longer GG-blocks which interacts with divalent cations such as Ca^2+^ allowing crosslinking in between the polymeric chains favoring more physical stable and flexible hydrogels, that could be used to formulate wound dressings or other biomedical devices that demand higher physical resistance in contrast alginates with higher M/G ratio could be associated with lower GG-block presences thus preventing crosslinking resulting in more brittle hydrogel which disfavors its application in biomedical products (Sanchez-Ballester et al., 2021; Ramos et al., 2018; Pritchard et al., 2017; Hoefer et al., 2015). Monomeric characterization of alginates begins with controlled depolymerization by alkaline hydrolysis to obtain short oligomers. These short oligomers can then be analyzed by HPLC, often coupled with refractive index detection, or by colorimetric assays such as the carbazole reaction. Furthermore, to assess polymer sequential analysis, spectroscopic methods such as High Resolution Nuclear Magnetic Resonance Spectroscopy (HNMR), which distinguishes the sequence and block distribution of M and G residues, and Fourier Transform Infrared Spectroscopy (FTIR-ATR) spectroscopy, which confirms the characteristic vibrational bands of carboxylate and C–O–C, allow the differential detection of each monomer by peak intensity. Nevertheless, NMR is considered the preferred method for alginate characterization due to its ability to assess monomer frequencies and the presence of dyads (MM, MG/GM, or GG) and even their possible triads (Díaz-Barrera et al., 2021; Ertesvåg, 2015; Gaytán et al., 2012; Peña et al., 2006).

Alginate has high biocompatibility and *in vivo* biodegradability (Martău et al., 2019), and it and its derivatives has been studied in diverse biomedical and biotechnological applications (Baldassarre et al., 2020; Hecht and Srebnik, 2016; Topuz et al., 2012; Yang et al., 2011), such as 3D cellular scaffolds in tissue engineering, wound dressings with better physical performance and delivery systems of bioactive compounds or as carriers for small therapeutic proteins (Qamar et al., 2024; Tan et al., 2023; Mallakpour and Mohammadi, 2022; Aguero et al., 2021; Mallakpour et al., 2021; Szekalska et al., 2016). The biomedical industry requires the use of materials with defined chemical compositions and of high purity. In the case of alginate, chemical characteristics such as molecular weight, the G/M ratio, and the presence of O-acetyl groups are highly relevant for its application in biomedical devices. In this context, different processes for obtaining and purifying alginate cannot ensure constant alginate quality in the previously presented terms (Sondermeijer et al., 2016; Ertesvåg, 2015; Bales et al., 2013; Langlois et al., 2009). Although most commercial alginates are of algal origin, various drawbacks are associated with their source, extraction, and refinement (Torres et al., 2019; Paredes-Juarez et al., 2014; Qi et al., 2009; Dusseault et al., 2006). In fact, Dusseault et al. (2006) demonstrated the presence of contaminants in various samples of algal and commercial alginates treated with different purification processes, obtaining products free of endotoxins, polyphenols, and proteins. Nevertheless, evidence from treatments with alginate beads has still revealed the presence of immunogenic proteins that compromise their therapeutic use. For this reason, it has been proposed that producing bacterial alginate in bioreactors using defined media would prevent the occurrence of these immunogenic proteins, while also ensuring the absence of endotoxins, polyphenols, and other contaminants. Futhermore, the chemical structure of algal alginate can vary due to climatic conditions, making it difficult to ensure a consistently well-characterized product. Additionally, the presence of phenolic compounds, proteins, lipids, and even mannitol bound to alginate requires additional purification steps such as precipitation which, although technically simple, can increase production costs and reduce overall process efficiency for many applications (food, pharma, and biomedical) (Bojorges et al., 2023; Saji et al., 2022). Finally, there is great concern about the ecological impact on marine fauna due to the harvesting of algal species (Bularz et al., 2022). These problems have been addressed by the study and elaboration of bioprocesses that involve the use of different biocatalysts, such as alginate-modifying enzymes, and fermentation with alginate-producing bacteria to ensure the production of high-quality alginates (Chuacharoen et al., 2022; Díaz-Barrera et al., 2021; Ertesvåg et al., 2017).

Although the properties of alginates have some limitations in terms of their G/M composition, it is interesting the current possibility of making *in vitro* modifications of alginate. Thus, *in vitro* modification of alginate can be carried out using alginate-lyase and C-5 mannuronan epimerases. Alginate-lyases are enzymes that catalyze the β-elimination of the 4-O-glycosidic bond between the inner residues of alginate, and the activity of this enzyme varies according to the neighboring residues of the bond. These enzymes can be used during structural sequencing of the polymer constituents; additionally, they are useful for obtaining bioactive molecules such as phenolic compounds from algae and help reduce alginate viscosity for dental applications. In addition, alginate epimerases catalyze the epimerization of M-residues to G-residues, which increases the numbers of G- and GM-blocks inside the alginate chain (Mollah et al., 2021). Alginate epimerases have been shown to produce alginates with high G contents and to modify the monomeric composition of alginates extracted from different algae to maintain a constant chemical composition (Tøndervik et al., 2020). Despite this background, even these processes are in the development stage and face problems with scaling up to enzymatic reactor quantities.

*Azotobacter vinelandii* is a bacterium capable of fixing atmospheric nitrogen and produces alginate during vegetative growth; during its differentiation process to a dormant state, it forms cysts (Gimmestad et al., 2009). *Azotobacter vinelandii* secretes alginate under vegetative growth conditions as part of the protection mechanism against oxygen inhibition of the nitrogenase complex (Inomura et al., 2017). During encystment, *A. vinelandii* produces an alginate layer that surrounds the dormant cell, called the central body. The two layers surrounding this cell are composed of alginate, alkylresorcinol and proteins (Rosales-Cruz et al., 2024). The outer layer, named the exine layer, is thin, rigid, and structured due to the inclusion of G-blocks by the action of C-5 mannuronan epimerases. In contrast, the inner layer (intine) has more MG- and MM-blocks, favoring an amorphous structure (Gawin et al., 2020).

The alginate biosynthetic pathways are similar in *A. vinelandii* and *Pseudomonas aeruginosa* and can be divided into four stages: precursor synthesis, polymerization, modification, and secretion (Dudun et al., 2022; Urtuvia et al., 2017; Maleki et al., 2017; Rehman et al., 2013; Franklin et al., 2011). Even though this polymer plays different roles in both microorganisms, the cluster of genes and regulatory events involved in alginate biosynthesis differ in each microorganism, affecting its chemical structure and thus its quality for biotechnological applications. These regulatory mechanisms are coordinated concerning the physiological state of the cell, which in turn depends on the environmental stimuli in which the cell develops (Núñez et al., 2022). Consequently, the complete description of the alginate biosynthetic pathway is complex, and the pathways must be separated into stages to better understand their mechanisms.

Furthermore, this review will analyze some strategies to optimize and improve alginate production. This review aims to present and discuss recent advances in biosynthesis, regulatory mechanisms, and the impact of oxygen transfer on alginate production.

## Alginate applications

Alginate is a well-known biopolymer with a variety of applications in different industries (Table 1). Traditionally, alginate has been applied mainly as a viscosifier in the food industry or because of its gelifying capacity. During the last few years, novel developments in the biomedical, pharmacological, and cosmetic industries have drawn attention to the vast importance of biopolymers. In this context, alginate has emerged as a useful resource in the development of new technologies, being most relevant as part of novel drug delivery systems and in the development of biomedical devices (Tomić et al., 2023).

**TABLE 1:** Applications of alginate.

Industry	Application	Chemical features	References
Pharmaceutic	Beads for probiotic protection	Low molecular weight (MMW = 40 kDa) and High G content (G/M rate = 2,3)	Ramos et al. (2018)
	Treatment for chronic respiratory disease	G-rich alginate oligosaccharide (MMW = 2.6 kDa, G/M rate = 9)	Pritchard et al. (2017)
	Soft tableting excipient	Sodium alginates of high MMW (180–400 kDa) and different G/M composition (G/M rate 0,43–2,6)	Schmid and Picker-Freyer (2009)
Biomedical	Bioink	High (75 kDa) and low (28 kDa) molecular weight comparison	Freeman and Kelly (2017)
	Hydrogel for myoblast adhesion and proliferation	High G (G/M rate = 1,5) and High M (G/M rate = 0,42) Alginates modified with covalent RGD peptide union	Rowley and Mooney (2002)
	Wound dressing	Bacterial alginate obtained from *A. vinelandii* (3,420 kDa, 59% w/v degree of acetylation; G/M rate = 0,67)	Hoefer et al. (2015)

The use of alginate as a biomaterial for developing biomedical devices is closely linked to its gelling properties. Although the simplest way to generate alginate hydrogels is to use divalent cations for cross-linking, several studies have proposed chemical modification techniques or the combination with other polymers to increase the degree of cross-linking between alginate chains. These strategies aim to enhance the structural stability and mechanical strength of the hydrogels while maintaining sufficient flexibility and conformability, which are essential for applications such as wound dressings (Ramos et al., 2018; Freeman and Kelly, 2017; Pritchard et al., 2017; Hoefer et al., 2015; Schmid and Picker-Freyer, 2009; Rowley and Mooney, 2002).

Compared with algal alginate, the resulting bacterial alginate had a greater molecular weight, ranging from 400 to 3,420 kDa, a higher G/M ratio (with a ratio from 2.3 to 9), and a high degree of acetylation (>50% w/w), explaining the greater water retention capacity and physical stability of the hydrogels made with bacterial alginate (Rashidzadeh et al., 2020; Pritchard et al., 2017; Hoefer et al., 2015; Schmid and Picker-Freyer, 2009). The authors developed alginate-based antibacterial hydrogel beads with a magnetic drug delivery response due to the inclusion of ferroferric oxide (Fe_3_O_4_) and silver (Ag) nanoparticles, which presented high antimicrobial activity against *Escherichia coli* and *Staphylococcus aureus* (Rashidzadeh et al., 2020).

In a pharmacological context, alginates have attracted attention as raw materials for many applications (Sanchez-Ballester et al., 2021). Alginate oligosaccharides are considered bioactive compounds with wide-ranging biological activities, such as antihypertensive, hypolipidemic, immunoregulatory, and hypoglycemic properties, among other possible applications (Xing et al., 2020). A clinical study revealed that daily and oral administration of 10 mg of 1 kDa alginate oligosaccharide reduced the progression of osteosarcoma by improving patients’ anti-inflammatory and antioxidant capacities and relieving cancer-related inflammation (Chen et al., 2017). Additionally, alginate is more extensively used in the pharmaceutical industry as a disintegrant in compressed tablets, tablet binders, tastemakers, and controlled-release matrices in soft tableting (Sanchez-Ballester et al., 2021). In this case, the primary structure of alginate becomes a determining factor for its drug release capability. In phosphate buffer (pH 7.2), the release of chlorpheniramine maleate and metronidazole is greater in M-rich alginate tablets. In contrast, G-rich alginates present a higher drug release rate in acidic media (Sriamornsak et al., 2007).

These studies reinforce the need to obtain high-quality alginates (with a G/M ratio greater than 2.3), with a well-defined chemical composition and a low presence of contaminants, for their application in pharmaceutical formulations or the manufacture of biomedical devices. The requirements of these industries cannot be met using algal alginate as a raw material, as its chemical features are commonly negatively affected during the separation and purification processes (Bojorges et al., 2023). Hence, other alternatives, such as bacterial production and enzyme-driven structural modification, have emerged as opportunities to obtain tailor-made alginates (Moradali et al., 2018). As both alternatives present advantages in obtaining high-end alginates, these processes participate in different stages during alginate production and should be assessed separately.

## Biosynthesis of alginate

**FIGURE 2:**
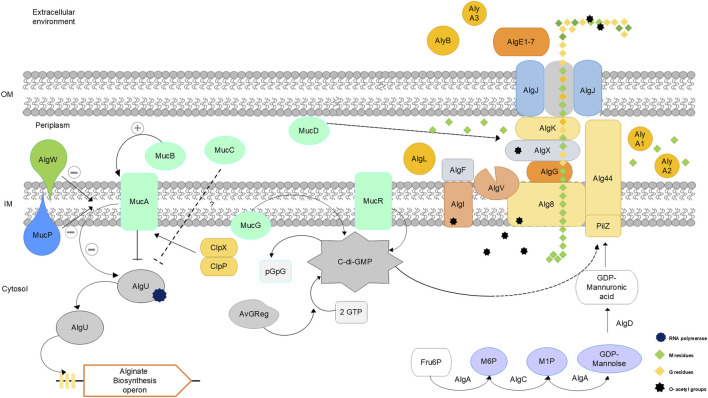
Schematic representation of alginate biosynthesis in *Azotobacter vinelandii*. Adapted from (Ahumada-Manuel et al., 2020; Urtuvia et al., 2017; Pacheco-Leyva et al., 2016; Ertesvåg, 2015; Flores et al., 2015; Hay et al., 2013).

The alginate biosynthesis process has been studied in *Azotobacter* sp. and *Pseudomonas* sp. (Núñez et al., 2022; Urtuvia et al., 2017; Hay et al., 2013; Franklin et al., 2011). The genes involved in biosynthesis are very similar, but some differ in their regulatory mechanisms. These genes are located in an operon that has been previously described: *algD*, *alg8*, *alg44*, *algK*, *algJ*, *algG*, *algX*, *algL*, *algI*, *algV*, *algF* and *algA* (Pacheco-Leyva et al., 2016; Hay et al., 2013; Chitnis and Ohman, 1993) (Table 2). A schematic representation of the biosynthesis of alginate synthesized by *A. vinelandii* is shown in Figure 2, integrating recent findings on this biosynthetic pathway.

**TABLE 2:** Proteins involved in alginate biosynthesis and regulation.

Protein	Description	Subcellular location	References
AlgA	Precursor synthesis. Phosphomannose isomerase/GDP-mannose pyrophosphorylase	Cytosol	Shinabarger et al. (1991)
AlgC	Precursor synthesis. Phosphomannomutase	Cytosol	Ye et al. (1994)
AlgD	Precursor synthesis. GDP-mannose dehydrogenase	Cytosol	Tatnell et al. (1994)
Alg8	Polymerization. Proposed glycosyltransferase/polymerase	Internal membrane	Remminghorst et al. (2009)
Alg44	Polymerization and post transcriptional regulation. c-di-GMP binding and response	Internal membrane	Remminghorst and Rehm (2006a)
AlgK	Export/structural role. Lipoprotein, Stabilizes AlgE Associated with periplasmic side of OM.	External Membrane	Keiski et al. (2010)
AlgJ	Export. OM porin	External Membrane	Whitney et al. (2011)
AlgG	M-G epimerization. Mannuronan C-5-epimerase	Periplasm	Franklin et al. (1994)
AlgL	Alginate lyase. Control MW, clear alginate from the periplasm	Periplasm	Jain and Ohman (2005)
AlgI	O-Acetylation	Internal Membrane	Franklin and Ohman (2002)
AlgV	O-Acetylation	Internal Membrane	Franklin and Ohman (2002)
AlgF	O-Acetylation	Periplasm	Franklin and Ohman (2002)
AlgX	O-Acetylation. Structural role. Sequesters MucD	Periplasm	Riley et al. (2013)
AlyA1	Alginate lyase	Periplasm	Gimmestad et al. (2009)
AlyA2	Alginate lyase	Periplasm	Gimmestad et al. (2009)
AlgE1-E7	*Azotobacter* extracellular Mannuronan C-5-epimerases	Extracellular	Ertesvåg et al. (2009)
AlyA3	Alginate lyase	Extracellular	Gimmestad et al. (2009)
AlyB	Alginate lyase uncharacterized	Extracellular	Gimmestad et al. (2009)
PilZ	PilZ domain of the copolymerase Alg44	Internal Membrane	Merighi et al. (2007)
AvGReg	C-di-GMP cytoplasmic DGC is produced from two GTP molecules by the action of (DGCs) with a GGDEF domain	Cytosol	Römling et al. (2013)
c-di-GMP	Ubiquitous second messenger bis (3 =, 5 =)-cyclic dimeric GMP.	Cytosol	Ahumada-Manuel et al. (2020)
MucG	Membrane protein which negatively affects alginate production and its molecular mass	Internal Membrane	Ahumada-Manuel et al. (2017)
MucR	Protein modulates a localized pool of c-di-GMP in proximity to Alg44 to control alginate production at post-translational level	Internal Membrane	Wang et al. (2015)
MucA	Anti σ factor. Negative regulator	Internal Membrane	Cezairliyan and Sauer (2009)
MucB	Stabilizes MucA. Negative regulator Periplasm	Periplasm	Martínez-Salazar et al. (1996)
MucC	Unclear regulatory role	Periplasm/Internal membrane	Boucher et al. (1997)
MucD	Homologous to *E. coli* serine protease DegP. Negative regulator. Associated with Alginate complex. Negative regulator	Periplasm	Hay et al. (2012), Wood and Ohman (2006)
AlgW	Homologous to *E. coli* serine protease DegS. Cleaves MucA. Positive regulator	Internal Membrane	Cezairliyan and Sauer (2009)
MucP	Homologous to *E. coli* RseP protease. Positive regulator, cleaves MucA.	Internal Membrane	Qiu et al. (2007)
ClpX/ClpP	Cytoplasmic proteases. Positive regulators, cleave MucA.	Cytoplasm	Qiu et al. (2008)
AlgU	Alternative σ factor homologous to *E. coli* σ^E^ global stress response factors. Positive regulator.	Cytosol	Mathee et al. (1997)
CydR	Repressor in cydA/B transcription. Homolog of Fnr.	Cytoplasm	Wu et al. (2000)
CydA/CydB	Genes encoding cytochrome bd terminal oxidase required for nitrogen fixation.	Cytoplasm	Wu et al. (2001)
AmrZ	Arc-like DNA-binding protein.	Cytoplasm	Baynham and Wozniak (1996)
AlgR	Two-component regulator (Cognate sensor is AlgZ/FimS). Positive regulator, binds to 3 regions in the *algD* promoter	Cytoplasm	S. Ma et al. (1998)
AlgB	NtrC-Family two-component regulator (Cognate sensor is KinB).	Cytoplasm	S. Ma et al. (1998)
RpoN	Alternating sigma factor RpoN (σ^54^)	Cytoplasm	Damron et al. (2012)
GacS/A	Two-component global regulatory system.	Internal Membrane	Castañeda et al. (2001), 2000
RsmA	Protein that binds directly to the algD mRNA, preventing its translation.	Cytoplasm	Manzo et al. (2012)
CbrA/CbrB	Two-component global regulatory system.	Internal Membrane	Quiroz-Rocha et al. (2017)
RpoS	Encodes the sigma factor S (σ^S^)	Cytoplasm	Castañeda et al. (2001)

Table 2 shows the proteins involved in alginate biosynthesis. Alginate biosynthesis can be divided into four steps: precursor synthesis, polymerization, modification, and secretion of alginate.

## Alginate precursor synthesis

The first step in the synthesis of alginate is the generation of the monomer mannose-6-phosphate (M6P). This step begins with the import of hexoses from the medium to the cytosol. Glucose undergoes enzymatic reactions *via* the Entner–Doudoroff pathway until the formation of glyceraldehyde-3-phosphate/di-hidroxyacetone 3-phosphate by the enzyme glyceraldehyde-3-phosphate dehydrogenase; these molecules are phosphorylated and then reconverted into fructose 6-phosphate (F6P). Through the coordinated action of three enzymes located in the cytosol, AlgA, AlgC and AlgD, four reactions are catalyzed, allowing the synthesis of the precursor GDP-mannuronic acid (Shinabarger et al., 1991; Ye et al., 1994; Tatnell et al., 1994). In this way, F6P is converted to M6P by the action of phosphomannose isomerase (AlgA, a bifunctional enzyme) and then converted to mannose-1-phosphate (M1P), which is catalyzed by phosphomannomutase (AlgC), followed by conversion to GDP-mannose, which is again catalyzed by AlgA (Zielinski et al., 1992). The last step is the conversion to GDP-mannuronic acid catalyzed by GDP-mannose dehydrogenase (AlgD), which is the irreversible step in the formation of the alginate precursor and involves the use of carbon atoms toward the formation of the polymeric chain (Hay et al., 2013; Gaona et al., 2004; Tavares et al., 1999).

## Polymerization

GDP-mannuronic acid is polymerized through two enzymes that are located inside the internal membrane (IM), glycosyltransferase/polymerase (Alg8) and copolymerase (Alg44), both of which are indispensable for the production of alginate (Urtuvia et al., 2017; Moradali et al., 2015; Hay et al., 2013; Oglesby et al., 2008; Galindo et al., 2007; Mejía-Ruíz et al., 1997). The process of polymerization and transit of the newborn alginate chain through the membrane has been partially described through studies in both *Pseudomonas aeruginosa* and *A. vinelandii* (Remminghorst et al., 2009; Oglesby et al., 2008; Remminghorst and Rehm, 2006b). The polymerization of alginate is regulated at the posttranslational level through the interaction of the PilZ domain of Alg44, which binds the secondary messenger bis-(3′-5′)-cyclic dimeric guanosine monophosphate (c-di-GMP), and allows the interaction with other proteins that are involved in the next steps of alginate polymerization and transport through the membrane (Ahumada-Manuel et al., 2020).

C-di-GMP is a global regulatory molecule involved in several cellular processes, such as motility, exopolysaccharide production and biofilm formation (Ahumada-Manuel et al., 2020; 2017; Römling et al., 2013). Intracellular levels of c-di-GMP are controlled by the activities of diguanylate cyclase with a conserved domain containing the GGDEF motif and by phosphodiesterases (PDEs) bearing the EAL or HD-GYP motif. Proteins involved in signal transduction cascades have c-di-GMP-binding domains, which include PilZ (Römling et al., 2013; Merighi et al., 2007).

## Modification, transport and secretion

During polymerization, the newborn chain of alginate is synthesized as a polymannuronic acid polysaccharide, which is translocated through the periplasm by the activity of some periplasmic proteins, such as AlgX, AlgG and AlgK (Riley et al., 2013; Keiski et al., 2010; Robles-Price et al., 2004; Jain et al., 2003; Franklin et al., 1994). During translocation, the poly-M alginate chain interacts with the protein complex composed of AlgI, AlgF, AlgV and AlgX, which presents acetyltransferase activity (Franklin and Ohman, 2002). This complex is capable of transferring an acetyl group from acetyl-CoA, which acts as a donor group, to the poly-M chain, which is the first event of chemical modification of the alginate chain, which is made on the C-2 and/or C-3 of the M-residues. AlgG has been identified as a protein with mannuronnan C-5 epimerase activity. Nevertheless, its activity is deficient in *A. vinelandii*, and its presence has a structural role in the alginate biosynthesis complex (Núñez et al., 2022; Ertesvåg, 2015). Therefore, the main event for the addition of G-residues into the alginate chain occurs after the secretion of the polymer into the extracellular medium, where the activity of seven different epimerases (AlgE1–7) in *A*. *vinelandii* cells can catalyze the epimerization of nonacetylated M-residues to G-residues (Stanisci et al., 2020).

AlgL is an alginate degradation protein with lyase activity that is encoded within the biosynthesis operon located in the periplasm. The role of AlgL is relevant for the viability of mucoid strains, exercising a maintenance function by degrading the alginate trapped in the periplasm (Jain and Ohman, 2005). AlgL can also actively control the polymer length and contribute to periplasmic translocation (Albrecht and Schiller, 2005; Bakkevig et al., 2005).

AlgK, together with AlgX, are structural proteins relevant for the process of alginate translocation through the periplasm; these proteins act as scaffolds for the assembly of functional alginate biosynthesis machinery (Keiski et al., 2010). In this context, AlgK (a lipoprotein) acts as an anchor for the protein complex in both the internal and external membranes.

The alginate chain, while transported through the periplasmic space, may undergo O-acetylation by the addition of hydroxyl O-2 and O-3 acetyl groups by four essential enzymes, AlgI, AlgV, AlgF, and AlgX, which can significantly alter the material properties of the resulting alginate (Fabich et al., 2012; Donati et al., 2009; Nivens et al., 2001). In addition, the O-acetylation of alginate is exclusive to bacterial alginates, which significantly increases the water retention capacity of alginate and the rigidity and structural homogeneity of alginate hydrogels, thus promoting the use of bacterial alginate as a biomaterial (Fabich et al., 2012; Nivens et al., 2001).

AlgI and AlgV are found in the IM of *A. vinelandii* cells, whereas AlgF and AlgX are found in the periplasm (Ertesvåg et al., 2017; Franklin and Ohman, 1996). AlgI transports acetyl-CoA groups from the cytoplasm to the periplasmic space where acetylation takes place. Acetyl groups are transferred by the actions of AlgF, AlgX, and AlgV to acetylate the M subunits in the alginate chain.

AlgG is responsible for the epimerization of nonacetylated M-residues in *P. aeruginosa* to convert them into G-residues, leading to the formation of alginate poly-MG (Maleki et al., 2017; Wolfram et al., 2014). In *A. vinelandii*, AlgG shares 60% structural homology with AlgG from *P. aeruginosa* and is essential in the alginate biosynthetic complex, although the main incorporation of G-residues in the polymer is made by soluble epimerases at the extracellular level (Nuñez et al., 2022).

*Azotobacter vinelandii* encodes six alginate lyases: alginate lyase (AlgL); mannuronan C-5 extracellular bifunctional epimerase and alginate lyase AlgE7 (which degrades the G-MM and G-GM bonds); three AlyA lyases, AlyA1, AlyA2 and AlyA3 (GM- and MG-cleavage); and an exolyase, AlyB (not characterized) (Ertesvåg, 2015; Setubal et al., 2009; Gimmestad et al., 2009; Ertesvåg et al., 2009). AlyA1 and AlyA3 are known to belong to the PL7 family of alginate lyases. AlyA1 and AlyA2 are related to growth.

AlgE1–7 are Ca^2+^-dependent enzymes that possess a regulatory domain (R-domain) sensitive to Ca^2+^, which regulates the activity of these enzymes through substrate affinity and catalytic modulation. These enzymes play different roles in *A. vinelandii,* as they are expressed under different conditions and have different catalytic activities in terms of the incorporation of diverse monomeric patterns through the alginate chain. For example, AlgE3 and AlgE1 can generate long MG patterns in the alginate chain, and AlgE6 can introduce long G-blocks; moreover, AlgE4 includes only individual G-residues, which promotes the generation of more flexible hydrogels because of the presence of short MG-blocks and GG-blocks. In terms of its physiological relevance, AlgE7 functions during cell germination from a cyst-dormant state because of its bifunctional activity (epimerase and lyase); AlgE3 and AlgE1 are related to vegetative growth, and AlgE2 is expressed during cyst differentiation (Moreno et al., 2018; Ertesvåg, 2015; Høidal et al., 2000).

In the last stage, after the polymeric chain is translocated through the periplasmic space, the porin protein AlgJ acts as a channel that allows the secretion of the alginate chain to the extracellular environment (Whitney et al., 2015; Baker et al., 2014; Hay et al., 2010).

## Regulation related to alginate biosynthesis and modification

The regulation of alginate biosynthesis is a well-conserved process in both *A. vinelandii* and *P. aeruginosa* and is also highly regulated because of the energy input and carbon flux required for its production, secretion and modification (Inomura et al., 2017; Pacheco-Leyva et al., 2016; Castillo et al., 2013). In *A. vinelandii*, the genes associated with alginate biosynthesis are clustered together in an operon away from the cluster of genes related to epimerization. Other biosynthetic genes, such as *algC,* or depolymerases, such as *AlyA1*, *AlyA2*, and *AlyA3,* can be found in different locations. The overall regulatory events related to alginate biosynthesis can be separated into three levels: transcriptional, posttranscriptional, and posttranslational (Núñez et al., 2022). In the case of alginate chemical modification, most of the regulation occurs at the transcriptional and posttranslational levels through events controlled by the ubiquitous secondary messenger c-di-GMP and by the action of global genetic regulators such as AlgR and RpoS (Ma et al., 1998; Mathee et al., 1997).

**FIGURE 3:**
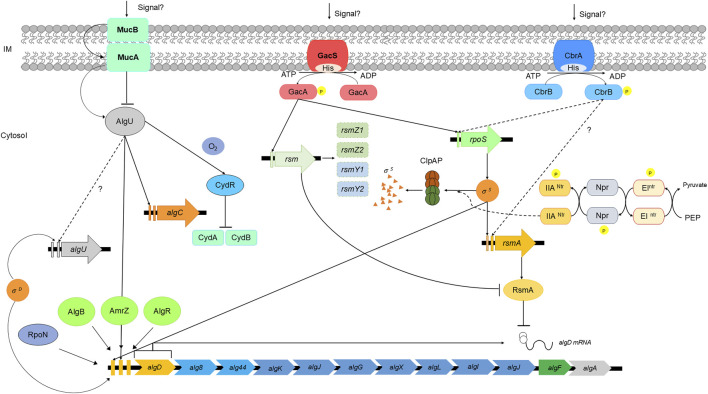
Schematic representation of alginate regulation in *Azotobacter vinelandii*. Adapted from (López-Pliego et al., 2020; 2018; Quiroz-Rocha et al., 2017; Romero et al., 2016; Pacheco-Leyva et al., 2016; Damron and Goldberg, 2012; Galindo et al., 2007; Castañeda et al., 2001).

A schematic representation of the regulation of alginate biosynthesis is shown in Figure 3.

The transcriptional regulation of alginate biosynthesis begins with the gene *algD,* which encodes the enzyme that mediates the carbon flux from sugars into the alginate precursor GDP-mannuronic acid. Three promoters have been found to exert positive effects on *algD* expression; two of these promoters are the sigma factors RpoS and AlgU, which also activate *algC* expression, and the third promoter activation factor (P3*algD*) remains unknown (Nuñez et al., 2022). In *A. vinelandii*, the sigma factor RpoS leads to a general response against oxidative stress and nutrient starvation and is essential during cell differentiation to the cyst dormant state. Moreover, the RpoE-like sigma factor AlgU also participates in the response against oxidative stress and is critical for alginate production during vegetative growth and encystment (Chowdhury-Paul et al., 2023; Martínez-Salazar et al., 1996). MucA is an antisigma factor that sequesters AlgU, preventing its action as a promoter of *algD* expression. Interestingly, AlgU and MucA, along with other antisigma factors, such as MucB, MucC*,* and MucD, are encoded within the same operon, suggesting a possible role in alginate biosynthesis regulation. Nevertheless, the regulatory role of MucC and MucD has not been elucidated to date (Núñez et al., 2022; Hay et al., 2012; Cezairliyan and Sauer, 2009; Wood and Ohman, 2006; Boucher et al., 1997; Martínez-Salazar et al., 1996). RpoS action is controlled by the response regulators RssB/RssC, which promote RpoS degradation through ClpXP during vegetative growth, acting as a repressor of the RpoS-related phenotype until the stationary phase (Rodríguez-Martínez et al., 2023).

After transcription, *algD* mRNA is regulated at the posttranscriptional level by the protein RsmA (CsrA). Rsm systems are RNA-binding proteins that act as posttranscriptional regulators belonging to the RsmA/CsrA family. In *A. vinelandii,* RsmA binds to the ribosome-binding site of *algD* mRNA, preventing its translation and thus negatively affecting alginate production. The expression of *algD* can be restored by the expression of small regulatory RNAs (*rsmZ1–8* and *rsmY*), and these sRNAs saturate the RsmA-binding site, allowing *algD* mRNA translation. The two-component system (TCS) GacA/GacS oversees the expression of *rsmZ1–7* and *rsmY*, and knocking out *gacA* or *gacS* prevents *algD* expression through *rsmA* action, annulling alginate biosynthesis (Rosales-Cruz et al., 2024; López-Pliego et al., 2020). Another TCS involved in alginate biosynthesis is the histidine kinase CbrA and its response regulator CbrB; these TCSs, together with the carbon catabolite repression system (Crc), control carbon flux in *A. vinelandii* and *Pseudomonas aeruginosa*. CbrA/CbrB mutants in the *A. vinelandii* wild-type strain present an alginate-overproducing phenotype. Other studies have shown that this TCS is required for high expression of *rsmA* and *rpoS* in *A. vinelandii* (Quiroz-Rocha et al., 2017; Manzo et al., 2012; Castañeda et al., 2000; 2001).

C-di-GMP is a universal secondary messenger in bacteria that controls many physiological processes, such as motility, pathogenesis, cellular differentiation, biofilm formation, and exopolysaccharide production (Valentini and Filloux, 2016). In *A. vinelandii* (as in *P. aeruginosa*), alginate polymerization through the polymerase complex Alg8/Alg44 is dependent on the presence of c-di-GMP through binding to the PilZ domain of Alg44. In the absence of this secondary messenger, alginate production is abolished; otherwise, its accumulation overstimulates alginate production (Merighi et al., 2007). The c-di-GMP pool is regulated through synthesis/degradation by specific enzymes. Diguanylate cyclases (DGCs) catalyze c-di-GMP production by the union of two GTP molecules at its active site, which is characterized by its GGDEF domain; commonly, DGCs contain an allosteric inhibition site that recognizes c-di-GMP; thus, these enzymes are inhibited by its product. Degradation of c-di-GMP occurs by the action of phosphodiesterases (PDEs) at specific EAL or HD-GYP domains, which catalyze the hydrolysis of this secondary messenger (Liang et al., 2022; Moradali and Rehm, 2020; Whitney et al., 2015). In the context of posttranslational regulation of alginate biosynthesis in *A. vinelandii*, AvGReg (DGC), MucR (DGC), and MucG (DGC-PDE) are the principal enzymes that control the c-di-GMP pool and thus promote or curb alginate production.

AvGReg is a globin-coupled sensor with DGC activity that directly binds oxygen and is considered the main contributor to the synthesis of c-di-GMP for alginate production. MucG can synthesize or degrade c-di-GMP due to the presence of both GGDEF and EAL domains (Ahumada-Manuel et al., 2017; Römling et al., 2013). Nevertheless, its PDE activity is the only one related to the inhibition of alginate production, as Δ*MucG* mutants can overproduce this polymer. Notably, MucG has a PAS domain that binds flavin adenine dinucleotide (FAD) cofactor, acting as a redox status sensor. This evidence suggests a relationship between respiratory metabolism, the redox status of the cell, and alginate production in *A. vinelandii*, as it has been shown that mutant strains affected by components of the respiratory chain show increased alginate production (Núñez et al., 2022; Ahumada-Manuel et al., 2020; Germani et al., 2020; Ahumada-Manuel et al., 2017). Interestingly, the PDE MucR is not essential for alginate polymerization, but it is essential during the encystment process, as *ΔMucR* mutants are unable to resist desiccation due to the lack of a well-assembled alginate capsule (Martínez-Ortiz et al., 2020).

*Azotobacter vinelandii* cysts are composed of two alginate layers named the intine and exine, and the guluronic acid contents of these two layers differ. The exine presents a higher G/M ratio than the intine does; consequently, the exine presents a rigid and structured shape in contrast to the intine, which presents a more amorphous structural characteristic (Martínez-Ortiz et al., 2020). In addition, Martínez-Ortiz et al. (2020) highlighted that MucR positively regulates the transcription of *algE1–6* genes through c-di-GMP signaling, probably through the transcriptional regulator FleQ, which presents binding sites in the regulatory region of the *algE1–7* genes. Additionally, MucR expression is regulated through AlgR, thus also being relevant in regulating alginate chemical modification, as Δ*AlgR* strains impair proper cyst coat organization. Moreover, c-di-GMP signaling also contributes to determining the molecular weight of the alginate chain, as an increase in the intracellular c-di-GMP pool promotes the production of alginates with higher mean molecular weights (MMWs), and AvGReg and MucG regulate this phenotype.

Epimerization of the alginate chain occurs at the extracellular level due to the action of the alginate epimerases AlgE1–7, which incorporate different patterns of MG- or GG-blocks of varying lengths depending on the individual activity of each epimerase. For example, AlgE4 modification incorporates long MG blocks through the alginate chain, whereas AlgE7 promotes long GG-blocks in the alginate chain (Petersen et al., 2023). The secretion of these epimerases requires the expression of the type 1 transport system *eexDEF*, which facilitates the transport of these enzymes through the membrane (Gimmestad et al., 2006).

The *EexDEF* transport system is encoded by an operon, and two promoters control its expression. P2_eexD_ is RpoS-dependent and is related to cyst differentiation, as RpoS also positively controls the expression of *AlgE1–7* genes, as AeRpoS (*ΔRpoS*-modified strain) can produce an alginate coat that is unable to resist desiccation, probably because of the lack of an organized structure capable of protecting dormant cells. P1_eexD_ is another promoter region that is an unknown activator. The guluronic content in alginate can be altered through changes in the oxygen concentration in the culture (Sabra et al., 2000), and P1_eexD_ is likely involved as a key control point, as it is necessary for epimerase excretion under other conditions not related to RpoS control (Moreno et al., 2018; Cocotl-Yañez et al., 2014).

## Oxygen transfer in alginate production

Oxygen plays a key role in the growth, maintenance and production of metabolites, such as during the synthesis of alginate (Flores et al., 2015). Studies have shown that alginate production by *A. vinelandii* is influenced by factors such as dissolved oxygen tension (DOT), agitation rate, aeration, and OTR (Ponce et al., 2021; Díaz-Barrera et al., 2021; Castillo et al., 2020; Díaz-Barrera et al., 2017; Flores et al., 2015; Díaz-Barrera et al., 2014; 2007).

Bacterial alginate production is a highly energy-demanding process that requires a high oxygen uptake rate (OUR) to synthesize this polymer (Castillo et al., 2020; Lozano et al., 2011). *Azotobacter vinelandii* has a high respiration rate (Oelze, 2000), which generates oxygen-limiting conditions during its growth (Ponce et al., 2021; Díaz-Barrera et al., 2021; 2014). Under these conditions, the dissolved oxygen content is almost zero (dO_2_/dt = 0), and according to the oxygen balance, the OTR is equal to the OUR, which is also known as microaerophilic conditions. Respiration can be assessed by measuring OTR, which remains constant during growth (Ponce et al., 2021; Díaz-Barrera et al., 2021; 2014; Garcia-Ochoa et al., 2010). These parameters are related to alginate production, alginate molecular weight, and the expression of biosynthetic genes (Díaz-Barrera et al., 2012).

In *A. vinelandii* cultures, a relatively high oxygen transfer rate (OTR) prevents the generation of high MMW alginates, as a decrease in the OTR promotes alginate polymerization through increased c-di-GMP accumulation (Ahumada-Manuel et al., 2020). Oxygen-sensitive enzymes can regulate c-di-GMP: AvGReg promotes c-di-GMP accumulation through DGC activity in the presence of the intracellular binding of O_2_, and MucG reduces the c-di-GMP pool through PDE activity, which is regulated through the binding of FAD as a redox state sensor (Noar and Bruno-Bárcena, 2018; Ahumada-Manuel et al., 2017; Peña C et al., 2011).

**FIGURE 4:**
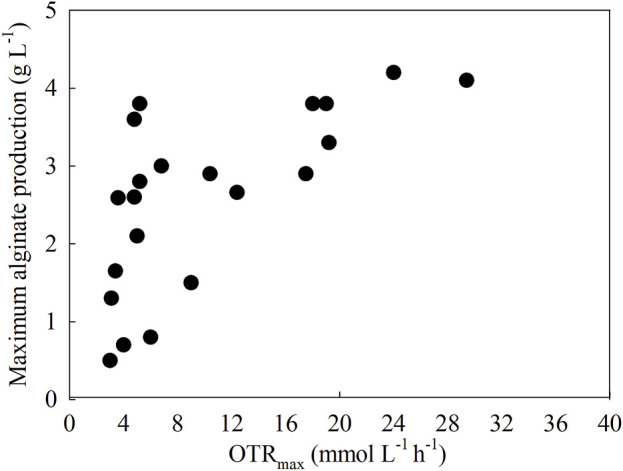
Relationship between the OTR and the alginate production in *A. vinelandii* under different culture conditions: batch cultures in flasks and bioreactors and continuous cultures.

One way to evaluate the role of oxygen in alginate production is to assess the effect of OTR on the maximum alginate concentration. Figure 4 shows a collection of scientific papers (n = 16) reporting data on OTR and alginate production under different culture conditions: batch cultures in flasks and bioreactors and continuous cultures.

The OTR_max_ is the highest rate at which oxygen is transferred from the gaseous phase to the liquid phase in a bioreactor and is limited by the aeration capacity and solubility of oxygen in the medium. It is a key parameter in aerobic culture systems to ensure an adequate oxygen supply (Maier and Büchs, 2001). The increase in OTR_max_ may be related to the increase in alginate concentration. At OTR_max_ values between 3 and 16 (mmol L^−1^ h^−1^), the value of the maximum alginate concentration increases from 0.8 to 3 (g L^−1^). Conversely, an increase in OTR_max_ from 16 to 30 (mmol L^−1^ h^−1^) does not significantly increase maximum alginate production. Two conclusions emerge from this relationship: an increase in OTRmax can modulate the maximum alginate production, which would help to generate defined culture strategies to produce and increase alginate production using a given OTR_max_. However, it could be argued that there is a limit at which OTR_max_ increases alginate production, so increasing OTR beyond the limit proposed in this review to improve alginate production is not an appropriate strategy.

## Effect of the oxygen transfer rate on the alginate molecular weight, degree of acetylation and G/M ratio

Several studies have measured OTR during *A. vinelandii* fermentation in flasks and bioreactors. These studies suggest that the OTR is related to the molecular weight of alginate. Some reports indicate that the highest molecular weight of alginate is obtained in batch cultures performed at a low OTR. However, as the OTR increases in cultures, the exact effect of OTR on the molecular weight of the alginate chain is still unknown.

**FIGURE 5:**
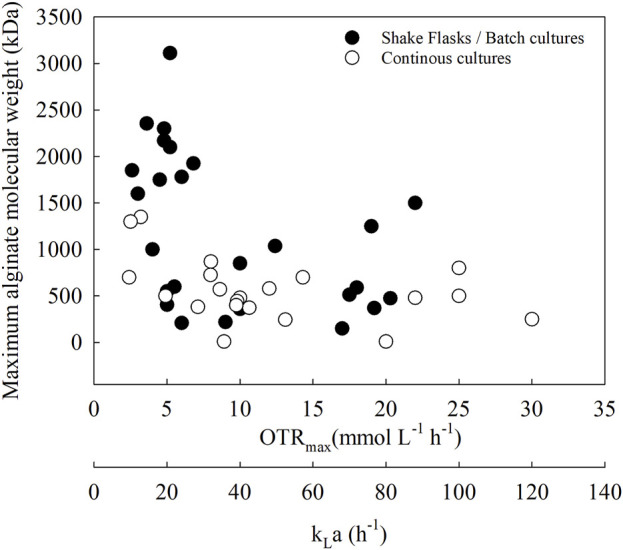
Relationship between the OTR and the alginate molecular weight in *A. Vinelandii* under different culture conditions: batch cultures in flasks and bioreactors and continuous cultures.

Figure 5 shows the relationship between OTR_max_ and the maximum alginate MMW. This figure shows a compilation of different studies (n = 16), establishing a relationship between OTR and different culture strategies and how this respiration parameter is related to alginate MMW in different *A. vinelandii* strains. All molecular weight data compiled in this analysis were determined using gel permeation chromatography (GPC) coupled to HPLC, ensuring consistency and comparability across studies (Díaz-Barrera et al., 2021; García et al., 2020; Díaz-Barrera et al., 2007).

At a low OTR_max_ (less than 10 mmol L^−1^ h^−1^), alginate greater than 1,000 kDa can be obtained in flasks, batch cultures (black circles), and continuous cultures (white circles). Conversely, in cultures with OTR_max_ values greater than 10 mmol L^−1^ h^−1^, alginate MMW does not change markedly and appears to be independent of OTR in all culture modalities.

Another relationship that has been established is the volumetric mass transfer coefficient (k_L_a) *versus* the maximum alginate MMW. Typically, k_L_a is a key parameter used for the optimization of oxygen availability in aerobic cultures and is also a parameter for scaling up to bioreactors (Garcia-Ochoa and Gomez, 2009).

In *A. vinelandii*, the high respiratory rate limits intracellular oxygen availability, thereby altering the OTRm_ax_ value. This, in turn, can modify the k_L_a of the culture, leading to changes in both alginate concentration and its mean molecular weight (MMW). Therefore, by adjusting k_L_a, it may be possible to control alginate size across different culture scales. Although no studies have yet used k_L_a as a scaling parameter to reproduce alginate MMW, some previous reports have managed to obtain similar alginate production at different scales using the OTR parameter in the culture, which is determined by k_L_a (Ponce et al., 2021; Díaz-Barrera et al., 2021; 2014). All this would help to produce a customized polymer according to the required application.

To understand the relationship between OTR and alginate production at the metabolic level, modified strains of *A. vinelandii* have been developed. A study carried out by García et al. (2020) revealed that the mutant strain AT9, with a truncated copy of the *mucG* gene (a negative regulator of alginate biosynthesis), produced alginates with greater MMWs (3,312 ± 150 kDa) than its parental strain ATCC9046 (2,170 ± 260 kDa) under microaerophilic conditions. Although both strains presented similar OTRs, the absence of MucG favored the formation of longer alginate chains, suggesting that PDE activity could regulate alginate polymerization in response to oxygen availability or the cellular respiratory status (García et al., 2020).

Other authors have highlighted the role of the secondary messenger c-di-GMP in regulating the production of exopolysaccharides in many bacteria. Recent studies have shown that the enzymes MucG, MucR and AvGReg control the biosynthesis and modification of alginate by affecting the levels of c-di-GMP, which is essential for activating the polymerase through the PilZ domain of Alg44. Furthermore, the strain *A. vinelandii* ATCC9046 presented greater accumulation of c-di-GMP, which is associated with the overproduction of alginate of higher molecular weight, regardless of changes in the sequence or expression of MucG (Moradali and Rehm, 2020; Wang et al., 2015).

As previously mentioned, c-di-GMP signaling is part of a control modulus that affects alginate production and its chemical characteristics in *A. vinelandii* cultures, and this signaling modulus can respond to changes in OTR. Figure 4 shows a plateau type of behavior that reaches its highest point at an OTR of approximately 16 (mmol L^−1^ h^−1^). These results could be attributed to the fact that c-di-GMP signaling is involved in the production of alginate, as an increase in OTR reduces the levels of c-di-GMP and, therefore, decreases the concentration of alginate; however, there is still a need to elucidate c-di-GMP.

As the OTR increases from approximately 4 to 16 (mmol L^−1^ h^−1^), c-di-GMP levels could decrease to a concentration sufficient for the binding and activation of Alg44, and an increase in alginate production can be reached through a more efficient distribution of carbon for alginate biosynthesis.

As the OTR increases until it reaches 16 (mmol L^−1^ h^−1^) or higher, the respiratory protection complex starts to increase the consumption of reducing power, which increases carbon flux through the TCA cycle to maintain NADH consumption by uncoupled electron transport terminals; consequently, more carbon is distributed into CO_2_ production over alginate. Finally, the alginate biosynthetic pathway could maintain this constant level of production, as it allows the production of two NADH molecules for every mannuronic acid molecule synthesized, which could help to maintain the redox balance of *A. vinelandii* under high-aeration conditions (García et al., 2018; Castillo et al., 2013).

In the opposite direction, as OTR_max_ is reduced, c-di-GMP levels increase, leading to the generation of alginates with higher MMW (Ahumada-Manuel et al., 2020). In agreement with these results, Figure 5 shows that longer alginate chains can be obtained in OTR_max_ ranges between 2 and 8 (mmol L^−1^ h^−1^), which could be related to control by this secondary messenger. Although a low OTR_max_ allows the generation of higher MMW alginates due to an increase in c-di-GMP levels, these conditions do not allow greater polymer production. An OTR_max_ of 5 (mmol L^−1^ h^−1^) is associated with an OTR of 1.5 (g L^−1^) of alginate, in contrast to conditions with higher oxygen availability, where the alginate concentration could increase to 4 (g L^−1^), with an OTR_max_ = 18 (mmol L^−1^ h^−1^).

As elevated c-di-GMP levels can alter many cellular processes, such as motility, differentiation, and polymer secretion, in this case, the reduced alginate production at low OTR_max_ can be explained through metabolic behavior. At low OTR_max_, oxygen limitation in *A. vinelandii* cultures negatively impacts carbon source intake due to the absence of oxygen as a terminal electron acceptor. This ultimately reduces the overall capacity of various biosynthetic pathways, including alginate production (Castillo et al., 2020). Notably, the viscosity of the broth increases due to the increased growth and secretion of alginates with high MMW values (>1,000 kDa), which could also exacerbate diffusional limitations from the medium to the intracellular level, affecting cellular growth and thus productivity.

In this scenario, modified strains such as *A. vinelandii* AT9 (mucG::mTn5; SpI) are useful tools for obtaining long alginate chains under high-OTR conditions (>16 mmol L^−1^ h^−1^) because of the ability of this strain to maintain elevated c-di-GMP levels without being significantly affected by OTR_max_. Finally, it becomes necessary to further investigate the relationship between the control modules of c-di-GMP at OTR_max_ values greater than 5 (mmol L^−1^ h^−1^), as previously reported by Ahumada-Manuel et al. (2020). To elucidate and validate these phenomena, alginate production and its modification are analyzed under conditions that favor these processes, such as non-diazotrophic cultures, alginate epimerization during vegetative growth, and the effects of global regulators on c-di-GMP levels, among others (Ahumada-Manuel et al., 2020).

In terms of the effect of oxygen on alginate production, oxygen influences the degree of alginate acetylation. The incorporation of acetyl groups confers amphiphilicity to alginate and improves the physical‒chemical properties of the formulated products (Tao et al., 2024; Wang et al., 2023).

Table 3 shows the acetylation degree under different conditions of cultivation (Medina et al., 2023; Jiménez et al., 2016; Castillo et al., 2013; Gaytán et al., 2012; Peña et al., 2006; Clementi et al., 1999; Peña et al., 1997). García et al. (2020) show that by using a mutant strain of *A. vinelandii* AT9 (mucG::miniTn5), in a bioreactor with sucrose as a carbon source and yeast extract as a nitrogen source, an increase in the OTR_max_ in the range of 3.6 to – 12.4 (mmol L^−1^ h^−1^) promoted cell growth and alginate production, generating an increase in the degree of acetylation (0.56%–2.13%) and, therefore, an increase in the viscosifying power. Castillo et al. (2013) evaluated the effects of DOT and the specific growth rate (μ) on the degree of acetylation in continuous cultures of *A. vinelandii* ATCC 9046, which were limited by glucose. They reported that high DOT (9%) resulted in a high degree of alginate acetylation (6.88%). In contrast, increasing μ had negative effects on polymer production and acetylation. At a high DOT content (9%) and low μ, there was a decrease in the respiration rate, suggesting that the flow of acetyl-CoA (the acetyl donor) was diverted toward alginate acetylation.

**TABLE 3:** Acetylation degree by *Azotobacter vinelandii* under different conditions of cultivation.

Strain	Carbon source	Nitrogen source	DOT (%)	Agitation rate (rpm)	Acetylation (%)	References
Shake flasks						
ATCC 9046	Glucose	Diazotrophy	NC	200	1.8	Castillo et al. (2013)
					4.7	
ATCN4	Sucrose	Diazotrophy	NC	200	4.2	Gaytán et al. (2012)
		Yeast extract			0	
ATCC 9046	Glucose	Diazotrophy	NC	200	4.7	Jiménez et al. (2016)
AT6					4.4	
ATCC 9046	Sucrose	Yeast extract	NC	200	0.7	Peña et al. (2006)
					0.9	
					1.2	
					1.4	
ATCC 9046	Sucrose	Yeast extract	NC	200	1	Peña et al. (1997)
Batch Cultures						
AT9	Sucrose	Yeast extract	NC	200	0.56	Medina et al. (2023)
				370	0.61	
				500	2.13	
ATCC 9046	Glucose	Diazotrophy	1	300	1.41	Jiménez et al. (2016)
AT6					1.54	
ATCC 9046	Sucrose	Yeast extract	3	300	3.6	Peña et al. (2002)
AT 268					3.6	
DM					2.6	
DSM 576	Glucose	(NH_4_)_2_SO_4_	NC	400	29.2	Clementi et al. (1999)

Another key parameter is the G/M ratio, which is crucial for defining the physical properties of these hydrogels (Díaz-Barrera et al., 2021). Table 4 shows various reports on the G/M ratio performed in flasks as well as in bioreactors using different types of *A. vinelandii* with different types of nitrogen sources (Díaz-Barrera et al., 2021; Gaytán et al., 2012; Kıvılcımdan Moral and Sanin, 2012; Peña et al., 2006; Clementi et al., 1999). Díaz-Barrera et al. (2021) evaluated the molecular weight and G/M ratio in batch cultures subjected to diazotrophic conditions at different OTRs. In this study, it was reported that the OTR affects the G/M ratio, as this ratio can be increased from 0.7 to 0.86 by increasing the OTR_max_ from 5 to 10.4 mmol L^−1^ h^−1^ through changes in the agitation rate. Gaytán et al. (2012) studied an alginate-overproducing strain called *A. vinelandii* ATCN4, which contains an insertion of the *nqrE* gene that encodes a subunit of the NADH-dependent sodium translocator complex: ubiquinone oxidoreductase (Na+-NQR). In this study, under nitrogen fixation conditions, this strain is capable of producing alginates with a high G/M ratio (close to 2); interestingly, when yeast extract was supplemented in the culture medium, this ratio increased to the highest G/M ratio (4.5) currently reported for alginates produced in *A. vinelandii* cultures.

**TABLE 4:** G/M ratio by *Azotobacter vinelandii* under different conditions of cultivation.

Strain	Carbon source	Nitrogen source	DOT (%)	Agitation rate (rpm)	G/M ratio	References
Shake flasks						
ATCN4	Sucrose	Diazotrophy	NC	200	2	Gaytán et al. (2012)
		Yeast extract			4.5	
ATCC 9046	Sucrose	Yeast extract	NC	200	0.78	Peña et al. (2006)
Batch Cultures						
ATCC 9046	Sucrose	Diazotrophy	NC	300	0.73	Díaz-Barrera et al. (2021)
				500	0.83	
				700	0.83	
ATCC 9046	Sucrose	Yeast extract	5	200	3.17	Kıvılcımdan Moral and Sanin (2012)
				400	2.50	
				700	3.63	
DSM 576	Glucose	(NH_4_)_2_SO_4_	NC	400	0.27	Clementi et al. (1999)

Recently, interest in the use of alginate, which has a relatively high gelling capacity and rigidity, for applications in the biomedical and pharmaceutical industries has increased. Therefore, it is necessary to develop strategies to ensure the production of alginates with a high G/M ratio through the production of alginate-overproducing mutant strains and different culture methods.

## Concluding remarks

This review highlights the importance of oxygen availability and its influence on intracellular c-di-GMP levels, regulating key enzymes that are involved in alginate polymerization and its modification. A high oxygen transfer rate (OTR) enhances the alginate concentration, whereas a lower OTR promotes the synthesis of alginate with a higher molecular weight and improved guluronic/mannuronic (G/M) ratios, which are critical for applications such as hydrogels and drug delivery systems. Future advancements should focus on engineering fermentation processes to fine-tune oxygen levels and use c-di-GMP signaling as a medication target because of its effect on the global regulation of alginate production and its chemical structure, which could favor the generation of alginates with better structural features. Genetic modifications targeting c-di-GMP metabolism could further enable precise control over alginate characteristics. By leveraging this regulatory pathway, it is possible to develop scalable and reproducible production systems that ensure consistency in biopolymer quality while maintaining economic and environmental sustainability.
